# Native Bees as Key Pollinators of Açaí (*Euterpe oleracea*) in the Central Amazon

**DOI:** 10.1007/s13744-026-01383-w

**Published:** 2026-04-16

**Authors:** João Victor Ladislau, Juliana Hipólito, Eduardo Freitas Moreira, Thiago Mahlmann, Marcio Luiz de Oliveira, Cristiane Krug

**Affiliations:** 1 Universidade Federal de Viçosa, Viçosa, Minas Gerais Brazil; 2 Instituto Nacional da Mata Atlântica, Santa Teresa, Espirito Santo Brazil; 3 National Institute of Science and Technology in Pollination: Knowledge, Conservation, and Sustainable Use of Pollinators (INCT INPol), Rio de Janeiro, Rio de Janeiro Brazil; 4 Universidade de São Paulo, São Paulo, São Paulo Brazil; 5 Instituto Nacional de Pesquisas da Amazônia, Manaus, Amazonas Brazil; 6 Instituto Nacional de Biodiversidad, Quito, Ecuador; 7 Embrapa Amazônia Ocidental, Manaus, Amazonas Brazil

**Keywords:** Amazon rainforest, Flower-visitor community, Meliponini, Pollination, Stingless bees

## Abstract

The açaí palm (*Euterpe oleracea*) is a key economic resource in the Amazon region. Despite this, studies on its floral visitors in Amazonas state remains scarce, outdated, lack species identifications and inferences about their role as pollinators. This study aimed to identify the floral visitor community and potential pollinators of an açaí crop in the central Amazon. From August 2018 to May 2019, we monitored the visits on 14 pistillate and 11 staminate inflorescences from 20 plants and calculated two indices: relative importance and pollen-transport efficiency, to identify the main potential pollinators. We collected 1656 insects from 111 species, mainly stingless bees (45.77%) and other native bees (33.32%). Ranking values identified *Trigona williana* Friese and *Partamona ferreirai* Pedro & Camargo as the principal contributors to pollen flow, followed by *Trigona dallatorreana* Friese and *Frieseomelitta trichocerata* Moure. This ranking method represents a key innovation of our study. Using these indices, we identify species contributing most to pollen flow while retaining information on the importance and efficiency of all taxa. Our findings reinforce the role of native bees in açaí pollination and provide potential target species for regional management.

## Introduction

In the Amazon region, most tree species that produce edible fruits rely on insects for cross-pollination (Klein et al. 2007; Paz et al. 2021), including açaí (*Euterpe oleracea* Martius, 1824), a tropical perennial palm tree native to the Amazon floodplain, naturally found in the Brazilian states of Amapá, Pará, Maranhão, and Tocantins (Vaz and Nabout 2016; Marques et al. 2024; Teixeira et al. 2025), which has recently become increasingly popular in the Brazilian market beyond its traditional producing states and recorded an increase in exports, playing a key role in the economy of the Amazon region (Silveira et al. 2023; Fonseca and Lima 2024; Pepper and Alves 2024). This popularization has been largely driven by the worldwide recognition of açaí as a “superfood” due to its health benefits (Yamaguchi et al. 2015; Barbosa and Carvalho Junior 2022; Silva et al. 2023).

The species (*E. oleracea*) is the main palm cultivated in Brazil, dominating crops in non-endemic states in the southeast, northeast, and north regions, including the state of Amazonas, which in 2023 produced approximately 105,221 tons (equivalent to US$ 40,184,048.83), despite the region having an endemic species (*E. precatoria* Martius, 1842) (D’Arace et al. 2019; IBGE 2025).

A wide variety of insect taxa have been recorded visiting açaí flowers in eastern Amazonia (Campbell et al. 2018); however, many of these include sporadic, opportunistic, and scavenger species that use floral resources without necessarily touching the reproductive structures or visiting both sexual phases of the inflorescences (Milet-Pinheiro and Schlindwein 2008; Alves-dos-Santos et al. 2016). Knowing the diversity of floral visitors and effective pollinators is key to developing management strategies for native biodiversity (Garibaldi et al. 2020), a factor that has already been identified as one of the main challenges in açaí production (Muto et al. 2020; Bezerra et al. 2020; Campbell et al. 2022).

There are few studies on the insect community associated with açaí (*E. oleracea*) in the state of Amazonas, such as the survey conducted by Gama (2004). However, they are mostly outdated, conducted mainly in the early 2000 s, and lack species identifications or inferences about the role of these visitors as pollinators.

Considering these points, the present study aimed to understand the community of floral-visitors insects associated with an açaí (*Euterpe oleracea*) cultivation and identify potential pollinators of the crop in the central Amazon region. In particular, to learn about this community and infer its effectiveness as pollinators, we asked the following questions: (1) What are the main insect taxa visiting the inflorescences of *Euterpe oleracea* in a plantation in central Amazonia, and how are they distributed between the male and female flowering phases? (2) Which floral visitors exhibit the highest potential to act as pollinators based on their contribution to pollen transfer?

## Material and Methods

### Study Area

**Fig. 1:**
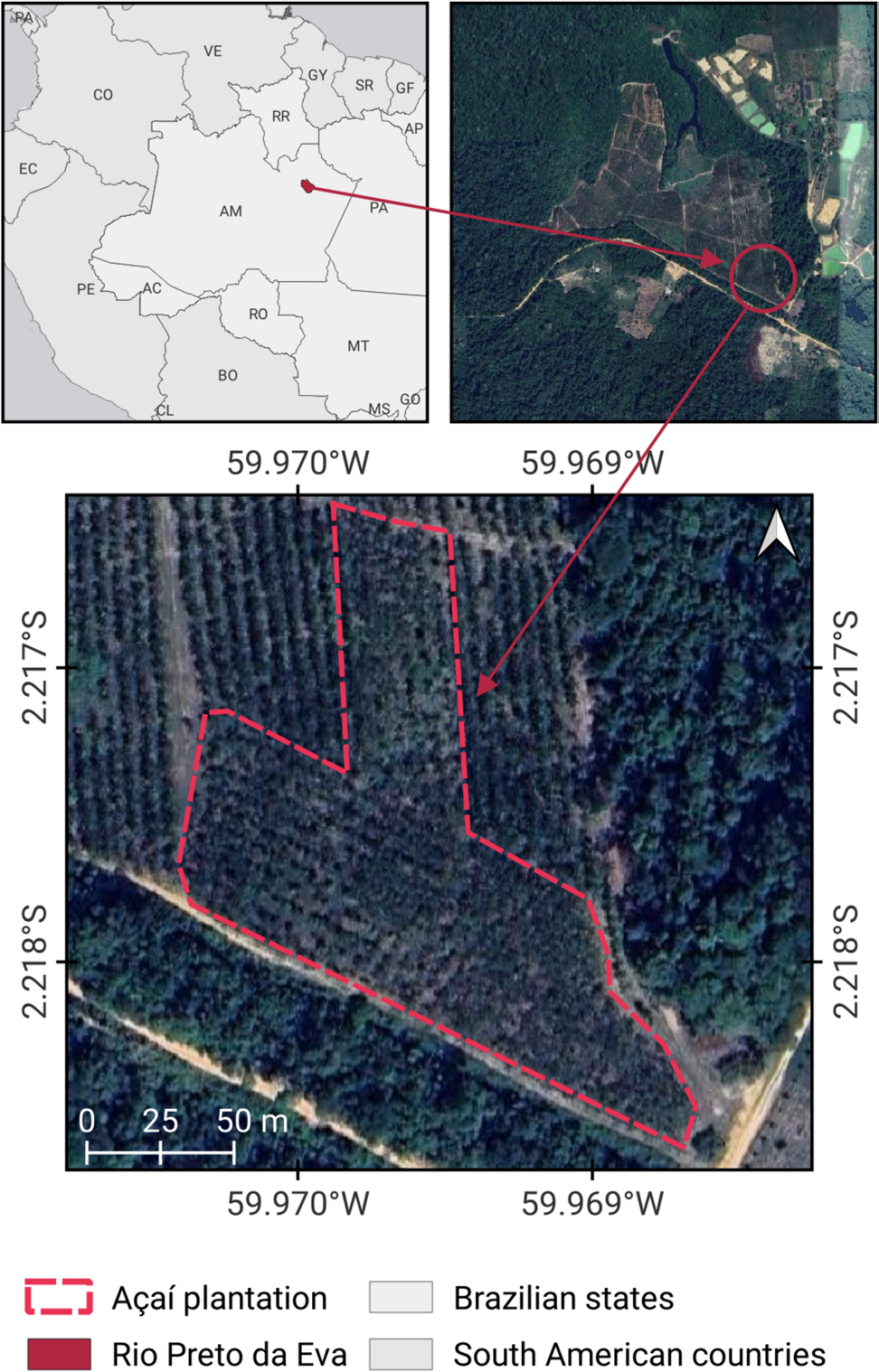
Location of the açaí *(Euterpe oleracea*) plantation, located on Highway BR 174, Km 960, branch ZF-6, Rio Preto da Eva, Amazonas, Brazil, prepared by João Victor Ladislau

The flower visitor community was sampled in a 13-ha experimental açaí crop located in Rio Preto da Eva, Amazonas state (2°13′S, 59°58′W) (Fig. 1). All individuals observed were young, three-year-old plants, approximately 3 m high, belonging to a monoculture area of BRS-Pará variety, a variety produced by Embrapa Western Amazon for the production of açaí fruits on non-flooded lands, characterized by small plants, early production, 3 years after planting, and first bunch at 112 cm high (Oliveira and Farias-Neto 2004) .

The region is surrounded by native primary forest typical of non-flooded humid tropical environments, featuring a canopy that reaches 30–37 m in height and an open understory with a high density of acaulescent palms (Henderson et al. 2019). The climate is humid tropical (type AM), with a mean annual temperature of 26.5 °C and rainy season between January and June, followed by a marked reduction in precipitation from July to September (Köppen 1936).

### Floral Biology of *Euterpe oleracea*

*Euterpe oleracea* is monoecious, dichogamous, and protandrous, with staminate flowers reaching anthesis before pistillate ones (Oliveira 2002; Oliveira et al. 2009). Reproductive maturity begins at approximately three years of age (Oliveira 2002; Oliveira et al. 2019). Flowering occurs year-round, with peaks between January and May, coinciding with the rainy season (Calzavara 1972; Jardim and Kageyama 1994; Oliveira 2002). Flowering starts with thermogenesis and spathe opening and proceeds in distinct male and female phases. The male phase lasts about 10–17 days, followed by a 3–6-day interval without flower opening and a shorter female phase of about nine days; total flowering duration averages 26 days (Oliveira et al. 2000; Oliveira 2002). Staminate flowers last 5–6 h, generally opening around 9:00 h, while pistillate flowers remain receptive for up to 24 h, predominantly opening during the day (Venturieri et al. 2014).

### Flower Visitor Samplings

Sampling was carried out between August 2018 and May 2019. In the field, 20 plants were monitored, comprising 14 pistillate inflorescences, and 11 staminate inflorescences. Captures were actively performed, using an entomological net. The insects were collected at the beginning and end of each observation period, repeating this procedure in three periods: 8:30–9:45 a.m., 10:15–11:30 a.m., and 2:00–3:15 p.m., covering the period of anthesis, dehiscence, and abscission of male flowers (Oliveira 2002), each inflorescence was monitored for 15 min, with 15-min intervals between each observation.

The collected material was deposited individually in perforated Eppendorf-type microtubes (the holes in the caps allowed the ethyl acetate vapor to enter and kill the insects), stored in plastic bags, and preserved in freezers (− 18 °C). The presence or absence of pollen on them was verified with the aid of a stereoscopic microscope. All specimens were mounted with No. 2 pins, dried in an oven at 40 °C for 72 h, identified by specialists in the groups, and deposited in the invertebrate collection of the National Institute for Amazonian Research.

### Data Analysis

The interaction network was used to visualize and quantify the structure of relationships between insect species and inflorescence phenophases, providing insights into interaction strength and specialization patterns, was characterized quantitatively (considering the abundance of each species as an indicator of the number of visits) and qualitatively (by the presence or absence of visiting species) using a weighted matrix. Rows represent insect species and columns represent inflorescence phenophases. The graphs were created using Gephi (0.9.2) and subsequently edited in Inkscape (1.4.2).

To estimate the importance and efficiency of floral visitor species as potential pollinators of the açaí crop in the field studied, the following indices were proposed:

1. The relative importance of each species for pollen flow (Imp), equal to the number of individuals of species with pollen captured in pistillate inflorescences (n_Ipf) divided by the total number of individuals with pollen recorded in pistillate inflorescences, considering all visitor species (n_pf):
  $$Imp=\frac{n\_Ipf}{n\_pf}$$
2. The efficiency of each species in pollen transport (Eff), equal to the number of individuals with pollen from species captured in pistillate inflorescences (n_Ipf) divided by the number of individuals with pollen from species captured in staminate inflorescences (n_Ipm):
  $$Eff=\frac{n\_Ipf}{n\_Ipm}$$

From these indices, a ranking value (*R*) was calculated by multiplying the relative importance values by the efficiency of each species:

R=Imp*Eff

We did not distinguish between pollen location; all visible pollen was included in the analyses.

## Results

**Fig. 2:**
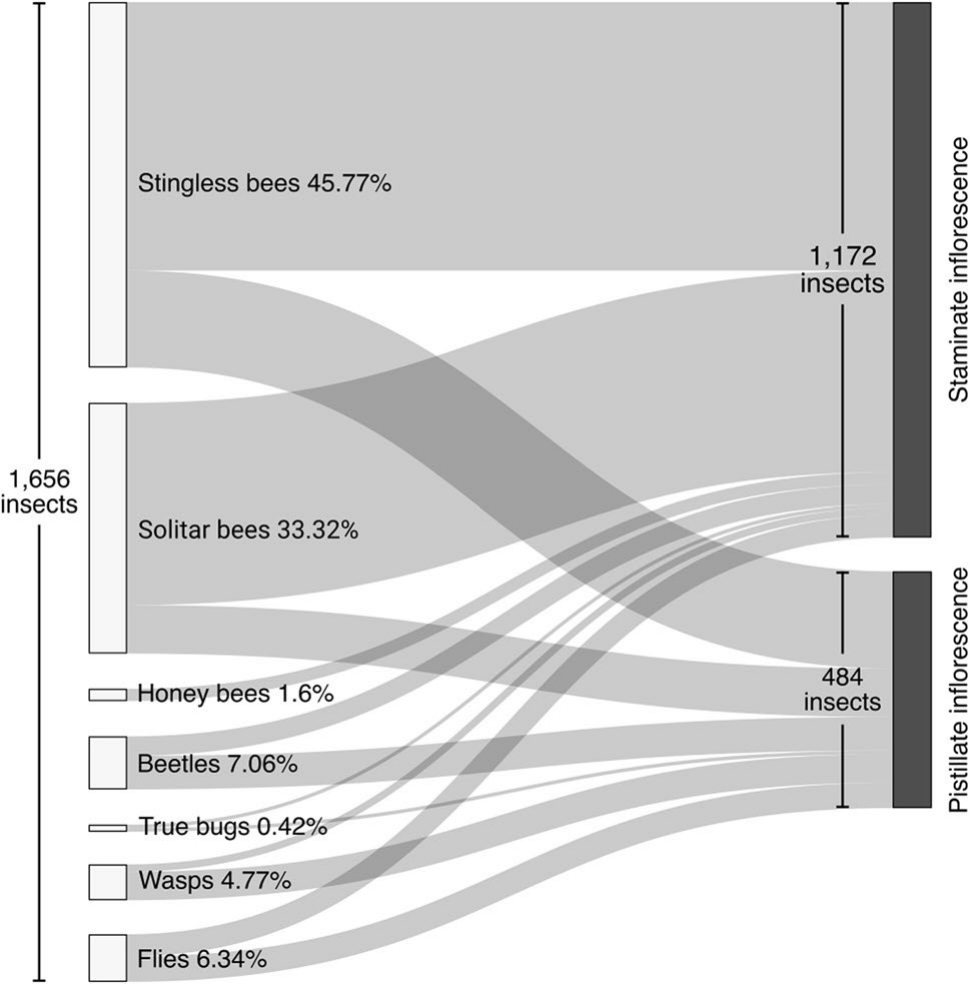
Sankey diagram summarizing floral visitation to staminate and pistillate inflorescences of *Euterpe oleracea*. Left-side nodes (white) represent the seven visitor groups, and right-side nodes (gray) represent the two inflorescence phenophases

One thousand six hundred fifty-six insects were collected from the inflorescences of açaí (*E. oleracea*), belonging to 111 taxa, with emphasis on stingless bees (Hymenoptera: Apidae: Meliponini; 45.77%), other native solitary bees (Apidae: Exomalopsini, Tapinotaspidini; Colletidae: Hylaeinae; Halictidae: Augochlorini, Halictini; 33.32%), beetles (Coleoptera; 7.06%), flies (Diptera; 6.34%), wasps (Vespidae: Epiponini, Polistini; 4.77%), and bugs (Heteroptera; 0.42%). 1,172 individuals of 73 species were collected in staminate inflorescences, compared to 484 of 82 species in pistillate inflorescences (Fig. 2). Forty-four species were observed in both sexual phases (Table 1).

**Table 1:** Flower-visitors of *Euterpe oleracea*, recorded from August 2018 to May 2019 in Rio Preto da Eva, Amazonas. N (abundance), % (percentage), Ip (number of individuals with pollen on their bodies), Stam inf (staminate inflorescences) and Pist inf (pistillate inflorescences)

Visitors	Visitors			Inflorescences	
	***N***	**%**	**Ip**	**Stam inf**	**Pist inf**
**Coleoptera**					
**Anthicidae**					
Anthicidae sp.	3	0.2		2	1
**Cantharidae**					
Cantharidae cf. sp.	1	0.1		0	1
**Chrysomelidae**					
Chrysomelidae sp. 1	1	0.1		1	0
Chrysomelidae sp. 2	1	0.1		1	0
Chrysomelidae sp. 3	1	0.1		0	1
Chrysomelidae sp. 4	1	0.1		1	0
Galerucinae cf. sp. 1	2	0.1		2	0
Galerucinae cf. sp. 2	8	0.5		8	0
Galerucinae sp. 3	2	0.1		1	1
**Coccinellidae**					
Coccinellidae sp.	3	0.2		2	1
**Curculionidae**					
Curculionidae sp. 1	81	4.9		19	62
Curculionidae sp. 2	5	0.3		4	1
Curculionidae sp. 3	1	0.1		0	1
Curculionidae sp. 4	1	0.1		0	1
Curculionidae sp. 5	1	0.1		0	1
**Histeridae**					
Histeridae sp.	1	0.1		0	1
**Mordellidae**					
Mordellidae sp. 1	1	0.1		0	1
Mordellidae sp. 2	1	0.1		0	1
**Nitidulidae**					
Nitidulidae sp.	2	0.1	1	2	0
**Hemiptera**					
Hemiptera spp.	5	0.3	1	3	2
**Scuteleridae**					
Scuteleridae spp.	2	0.1		2	0
**Hymenoptera**					
**Apidae**					
**Apini**					
*Apis mellifera* Linnaeus, 1758	27	1.6	18	27	0
**Exomalopsini**					
*Exomalopsis minor* Schrottky, 1910	11	0.7	10	10	1
**Meliponini**					
*Camargoia camargoi* Moure, 1989	2	0.1	2	1	1
*Frieseomelitta trichocerata* Moure, 1990	16	1	4	6	10
*Melipona amazonica* Schulz, 1905	2	0.1	2	2	0
*Melipona flavolineata* Friese, 1900	2	0.1	2	2	0
*Melipona illustris* Schwarz, 1932	1	0.1	1	1	0
*Oxytrigona obscura* (Friese, 1900)	1	0.1		1	0
*Paratrigona euxanthospila* Camargo & Moure, 1994	9	0.5	7	6	3
*Paratrigona melanaspis* Camargo & Moure, 1994	4	0.2	2	3	1
*Partamona* sp.	1	0.1	1	1	0
*Partamona auripennis* Pedro & Camargo, 2003	21	1.3	16	15	6
*Partamona ferreirai* Pedro & Camargo, 2003	21	1.3	19	4	17
*Partamona mourei* Camargo, 1980	1	0.1	1	0	1
*Partamona pearsoni* (Schwarz, 1938)	2	0.1	2	0	2
*Partamona vicina* Camargo, 1980	13	0.8	5	10	3
*Plebeia* sp. 1	4	0.2	3	4	0
*Plebeia* sp. 2	1	0.1		1	0
*Plebeia* sp. 3	15	0.9	5	12	3
*Plebeia* sp. 4	2	0.1	1	1	1
*Ptilotrigona lurida* (Smith, 1854)	10	0.6	7	7	3
*Tetragona goettei* (Friese, 1900)	6	0.4	3	4	2
*Trigona* sp.	1	0.1		1	0
*Trigona* sp. nov. (Ribeiro in press)	17	1	10	12	5
*Trigona albipennis* Almeida, 1995	3	0.2	2	2	1
*Trigona amazonensis* (Ducke, 1916)	1	0.1		0	1
*Trigona cilipes* (Fabricius, 1804)	1	0.1		0	1
*Trigona compressa* Latreille, P. A. (1811)	2	0.1		0	2
*Trigona dallatorreana* Friese, 1900	508	31	328	415	93
*Trigona dimidiata* Smith, 1854	1	0.1		0	1
*Trigona guianae* Cockerell, 1910	6	0.4	4	2	4
*Trigona williana* Friese, 1900	83	5	56	32	51
*Trigonisca* sp.	1	0.1		1	0
**Tapinotaspidini**					
*Paratetrapedia basilaris* Aguiar & Melo, 2011	1	0.1		0	1
*Paratetrapedia connexa* (Vachal, 1909)	4	0.2	1	3	1
*Paratetrapedia duckei* (Friese, 1910)	5	0.3		3	2
**Colletidae**					
**Hylaeinae**					
*Hylaeus* sp.	1	0.1		0	1
**Halictidae**					
**Augochlorini**					
*Augochlora* sp. 1	3	0.2	1	3	0
*Augochlora* sp. 2	2	0.1	1	2	0
*Augochlora* sp. 3	1	0.1	1	1	0
*Augochloropsis* sp. 1	4	0.2	2	3	1
*Augochloropsis* sp. 2	8	0.5	6	8	0
*Augochloropsis* sp. 3	5	0.3	3	5	0
*Neocorynura* sp. 1	3	0.2	1	2	1
*Neocorynura* sp. 2	16	1	12	13	3
*Neocorynura* sp. 3	1	0.1		0	1
*Pereirapis semiaurata* (Spinola, 1851)	1	0.1		1	0
*Temnosoma* sp.	1	0.1		1	0
**Halictini**					
*Habralictus* sp. 1	202	12	24	152	50
*Habralictus* sp. 2	290	18	24	247	43
*Lasioglossum* (*Dialictus*) sp.	4	0.2	1	1	3
**Vespidae**					
**Epiponini**					
*Agelaia angulata* (Fabricius, 1804)	1	0.1		0	1
*Agelaia lobipleura* (Richards, 1978)	1	0.1		0	1
*Brachygastra scutellaris* (Fabricius, 1804)	1	0.1		0	1
*Clypearia sulcata* (de Saussure, 1853)	1	0.1		1	0
*Polybia* sp.	1	0.1		0	1
*Polybia bistriata* (Fabricius, 1804)	2	0.1		1	1
*Polybia liliacea* (Fabricius, 1804)	3	0.2		1	2
*Polybia occidentalis* (Olivier, 1792)	7	0.4		4	3
*Polybia procellosa dubitata* Ducke, 1910	1	0.1		1	0
*Polybia rejecta* (Fabricius, 1798)	8	0.5		1	7
*Polybia scutellaris* (Write, 1841)	1	0.1		0	1
*Protopolybia chartergoides* (Gribodo, 1892)	50	3	2	6	44
**Polistini**					
*Polistes geminatus* Fox, 1898	1	0.1		0	1
*Polistes versicolor* (Olivier, 1791)	1	0.1		1	0
**Diptera**					
**Agromyzidae**					
Agromyzidae sp.	1	0.1		0	1
**Asilidae**					
Asilidae sp. 1	1	0.1		0	1
Asilidae sp. 2	1	0.1		0	1
**Calliphoridae**					
Calliphoridae sp.	2	0.1		0	2
**Chloropidae**					
Chloropidae sp.	36	2.2	1	17	19
**Lonchaeidae**					
Lonchaeidae sp.	2	0.1		1	1
**Phoridae**					
Phoridae sp.	3	0.2		0	3
**Sarcophagidae**					
Sarcophagidae sp. 1	4	0.2		2	2
Sarcophagidae sp. 2	4	0.2		0	4
Sarcophagidae sp. 3	1	0.1		0	1
Sarcophagidae sp. 4	1	0.1		0	1
**Scatopsidae**					
Scatopsidae sp.	1	0.1		0	1
**Sciaridae**					
Sciaridae sp.	1	0.1		0	1
**Syrphidae**					
Syrphidae sp. 1	15	0.9	1	11	4
Syrphidae sp. 2	7	0.4		4	3
Syrphidae sp. 3	1	0.1		1	0
Syrphidae sp. 4	4	0.2		1	3
Syrphidae sp. 5	1	0.1		0	1
**Tachinidae**					
Tachinidae sp. 1	1	0.1		0	1
Tachinidae sp. 2	1	0.1		0	1
Tachinidae sp. 3	1	0.1		1	0
Tachinidae sp. 4	1	0.1		0	1
Tachinidae sp. 5	1	0.1		0	1
Tachinidae sp. 6	2	0.1		1	1
**Ulidiidae**					
Ulidiidae spp.	12	0.7		10	2
	1656	100	594	1141	515

**Fig. 3:**
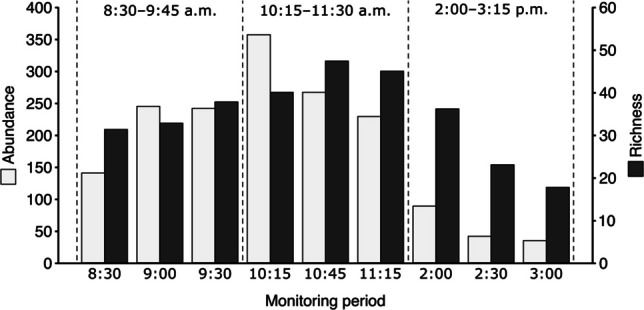
Average abundance and richness of *Euterpe oleracea* floral visitors across three monitoring periods

The morning monitoring period was the period of greatest activity of the visitor’s community in the crop. Both abundance and richness peaked between 10:00 and 11:00 a.m., when activity began to decline sharply. Despite the rapid decline in abundance, richness declined less sharply. Ninety-seven of the 111 species (81.8%) sampled were captured between 8:30 and 11:30 a.m. (Fig. 3).

**Fig. 4:**
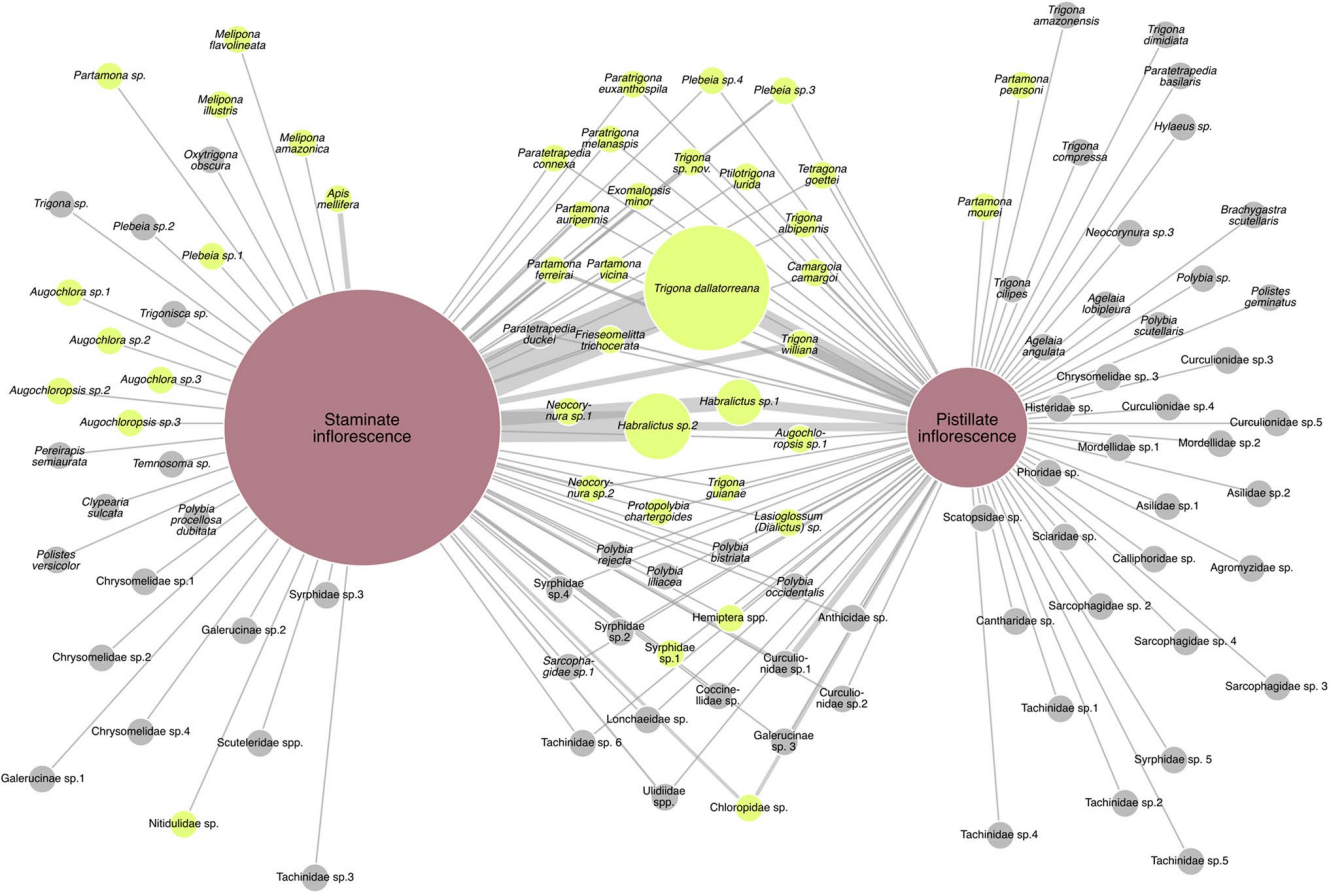
Interactions between floral visitor species and the staminate and pistillate açaí inflorescences. Insect node size = total abundance of each species; inflorescence node size = combined abundance of all species collected on them. Link width = relative abundance of each species per inflorescence type. Green = insects with pollen, gray = without pollen, purple = inflorescences

Pollen grains were found on the bodies of 42 of the 111 species (594 individuals, 35.6% of the total sampled) (Table 1). Twenty-nine of these species were recorded visiting both sexual phenophases of the açaí inflorescences (Fig. 4). Fifteen had an abundance of 10 or more individuals with pollen on their bodies, with 11 of them also captured in pistillate inflorescences. Bees were the only group represented by species with more than one individual carrying pollen. Other groups observed had only four species with pollen on their bodies, all with an abundance of two individuals or less (Table 2).

**Table 2:** Most abundant floral visitors. N (abundance), Ip (number of individuals with pollen on their bodies), Ipf (individuals with pollen in pistillate inflorescences), and Ipm (individuals with pollen in staminate inflorescences)

Visitors	*N*	Ip	Ipp	Ips
*Trigona dallatorreana* Friese, 1900	508	328	29	299
*Habralictus* sp. 2	290	24	-	24
*Habralictus* sp. 1	202	24	-	24
*Trigona williana* Friese, 1900	83	56	40	16
*Protopolybia chartergoides* (Gribodo, 1892)	50	2	1	1
*Apis mellifera* Linnaeus, 1758	27	18	-	18
*Partamona auripennis* Pedro & Camargo, 2003	21	16	4	12
*Partamona ferreirai* Pedro & Camargo, 2003	21	19	15	4
*Trigona* sp. nov. (Ribeiro in press)	17	10	1	9
*Frieseomelitta trichocerata* Moure, 1990	16	4	3	1
*Neocorynura* sp. 2	16	12	3	9
*Plebeia* sp. 3	15	5	-	5
*Partamona vicina* Camargo, 1980	13	5	1	4
*Exomalopsis minor* Schrottky, 1910	11	10	1	9
*Ptilotrigona lurida* (Smith, 1854)	10	7	2	5
*Paratrigona euxanthospila* Camargo & Moure, 1994	9	7	2	5

The ranking values (*R*) indicated that *Trigona williana* and *Partamona ferreirai* were the most effective potential pollinators of açaí in the monitored cultivation, as they showed values an order of magnitude higher for both importance (Imp) and efficiency (Efi) compared to the other pollen-carrying visitor species. Two other species, *Trigona dallatorreana* Friese, 1900 and *Frieseomelitta trichocerata* Moure, 1990, had high importance and efficiency values, respectively. Other species also have individuals with pollen on their bodies recorded in pistillate inflorescences, but with much lower importance and efficiency indices than the species already mentioned (Table 3).

**Table 3:** Potential pollinators captured in pistillate inflorescences of *Euterpe* *oleracea*. Imp (relative importance of each group for the relevant pollination flow), Eff (efficiency of each species in pollen transport), and *R* (ranking)

Potential pollinators	Imp	Eff	*R*
*Trigona williana* Friese, 1900	0.367	2.5	0.9174
*Partamona ferreirai* Pedro & Camargo, 2003	0.138	3.8	0.5160
*Frieseomelitta trichocerata* Moure, 1990	0.028	3.0	0.0825
*Trigona dallatorreana* Friese, 1900	0.266	0.1	0.0258
*Trigona guianae* Cockerell, 1910	0.018	1.0	0.0183
*Partamona auripennis* Pedro & Camargo, 2003	0.037	0.3	0.0122
*Augochloropsis* sp. 1	0.009	1.0	0.0091
*Camargoia camargoi* Moure, 1989	0.009	1.0	0.0091
*Melipona flavolineata* Friese, 1900	0.009	1.0	0.0091
*Neocorynura* sp. 2	0.028	0.3	0.0091
*Paratrigona euxanthospila* Camargo & Moure, 1994	0.018	0.4	0.0073
*Ptilotrigona lurida* (Smith, 1854)	0.018	0.4	0.0073
*Partamona vicina* Camargo, 1980	0.009	0.3	0.0022
*Exomalopsis minor* Schrottky, 1910	0.009	0.1	0.0010
*Trigona* sp. nov. (Ribeiro in prep.)	0.009	0.1	0.0010

## Discussion

### Composition of the Floral Visitor Community

Previous studies on the community of floral visitors to palm trees of *E. oleracea* in plantations in the eastern Amazon have highlighted the importance of generalist (non-specialized) species in the pollen flow of this group (Bezerra et al. 2020; Campbell et al. 2023). In the present study, we found the presence of several insect groups, including native solitary and eusocial bees, wasps, flies, beetles, and bugs visiting the inflorescences of *E. oleracea*. Our data suggest, under the circumstances and limitations defined in the present study, that native bees, represented here mainly by stingless bees, are a group of unquestionable importance in the pollen flow of açaí in central Amazon.

Hymenoptera was the main group sampled, representing 86.17% of the total insects sampled, especially native bees of the Meliponini and Halictini tribes (represented mainly by the genera *Trigona* and *Habralictus*, respectively). These results are in line with the recent findings of Bezerra et al. (2020) and Campbell et al. (2023) in açaí palm trees in the Belém-PA region, where 51% of the total insects sampled belonged predominantly to these groups. According to Barfod et al. (2003), bees are often found in species that allow for extensive pollen foraging. In açaí, the abundant number of small flowers (on average 18,478 male and 4857 female) grouped in dense inflorescences, with abundant resources and relatively large, easy-to-handle anthers, may be associated with the majority presence of these taxa in the study (Borja-Rentería et al. 2024).

### Daily Variation in Visitor Activity

Visitor community activity was highest during the first observation intervals (8:30 to 11:30 a.m.). Hymenoptera was the richest and most abundant taxon in all three periods. These results agree with Bezerra et al. (2020), who observed not only the predominance of interactions in the early periods of the day, but also a significant decrease in the number of pollen grains collected after the beginning of the afternoon period in their studies. The volume of nectar and sugar concentration in pistillate flowers of *E. oleracea* peak around noon, declining as the hours pass (Venturieri et al. 2014), and these conditions are associated with the reduction in floral resources observed by Bezerra et al. (2020) and the specificity due to narrow temperature variations in some bee species (Lima et al. 2018), the main constituent group of our observations, may be related to the sharp decrease in abundance after 1:00 p.m. in our study.

### Contribution of Taxa to Pollen Flow

Previous studies on pollination of the palm species of the genus *Euterpe* have already demonstrated the importance of various taxa in the pollen flow of the group (Marques-Souza et al. 1996; Wendt et al. 2011; Dorneles et al. 2013; Campbell et al. 2018, 2022, 2023; Bezerra et al. 2020; Zamudio et al. 2021). However, only native bees of the tribe Meliponini were captured on pistillate inflorescences with pollen on their bodies in our study. Other native bees, especially the tribe Halictini (represented here mainly by two species of the genus *Habralictus*), were also captured in both sexual phases, but none of the individuals captured on pistillate inflorescences had pollen on their bodies. Honey bees, *Apis mellifera*, although reported as one of the main visitors of pistillate flowers in cultivated areas in Pará State (Maués et al. 2024), were observed in our study in Amazonas State exclusively on staminate flowers under the conditions evaluated. The other groups captured in the study have only one species each with pollen records, all equally captured only on staminate inflorescences.

Our results revealed a predominance of Hymenoptera, particularly native bees, visiting the inflorescences of *Euterpe oleracea*. This pattern is consistent with observations from açaí crops in Pará state, where bees were also reported as the most representative floral visitors (Bezerra et al. 2020; Campbell et al. 2023; Maués et al. 2024). Previous studies had already emphasized the contribution of Meliponini to the pollination of this species, highlighting their importance as effective pollinators in cultivated systems (Henderson et al. 2000; Venturieri et al. 2014; Venturieri 2015).

These findings contrast with earlier assumptions that pollination in *E. oleracea* would be primarily associated with beetles, particularly Curculionidae, a family commonly linked to the pollination of tropical palms (Küchmeister et al. 1997; Listabarth 2001). In our observations, the most frequently recorded curculionid species was more often associated with pistillate inflorescences than with staminate ones. However, none of the individuals captured on staminate flowers carried pollen on their bodies. Although this pattern differs from Campbell et al. (2023), who reported curculionids as rarely occurring on pistillate inflorescences, both studies indicate a minor participation of this group in the pollination process of açaí under the respective study conditions.

Most tropical palms are pollinated by specialist taxa, mainly beetles of the Curculionidae family (Souza et al. 2018). The group is associated with palms with inflorescences of numerous small flowers concentrated in clusters, usually with nocturnal anthesis and protogyny (female flowers opening first) since they carry out their reproductive processes within the inflorescences, taking advantage of the thermogenesis performed by numerous species of Arecaceae for the maturation of juveniles, with their larvae feeding on floral tissue and their adults on pollen (Listabarth 2001; De Medeiros et al. 2019). *E. oleracea*, however, have diurnal anthesis, are protandrous (male flowers open first) and have greater floral resources and sugar concentration in the morning (8:00–11:00), these factors are more aligned with the bees’ period of activity, since beetles peak during the night (Küchmeister et al. 1997; Listabarth 2001; Venturieri et al. 2014), which may explain the predominance of Hymenoptera species in our study.

Bees of the tribe Halictini, represented here by two species of the genus *Habralictus*, accounted for 29.7% of our samples. This genus had previously been recorded on *E. oleracea* inflorescences in studies by Bezerra et al. (2020), Paz et al. (2021), and more recently by Campbell et al. (2023). These bees are known for carrying large amounts of pollen on plumose bristles located on the ventral side of the thorax and coxae. The pollen stored here is not mixed with nectar and is thus easily deposited on stigmas during floral visits, making them excellent pollinators for many plants (Parker et al. 2015; Stavert et al. 2016; Russell et al. 2021). However, despite their high frequency in our study, no specimens with pollen on their bodies were captured on pistillate flowers, even though females of both species were frequently collected on pistillate inflorescences.

### Potential Pollinators and Ranking

Results from the species ranking based on visitation frequency and pollen transport efficiency between inflorescence types highlighted two species, *T. williana* and *P. ferreirai*, as the most important contributors to pollen flow in the studied crop, due to their high visitation frequency and high importance and efficiency values compared to other species. Two other species, *T. dallatorreana* and *F. trichocerata*, also ranked well. However, unlike the previously mentioned species, *T. dallatorreana* (the most abundant in the study) showed low pollen transport efficiency, as most pollen-carrying individuals were collected on staminate inflorescences. Meanwhile, *F. trichocerata*, despite its high efficiency values, was one of the least sampled species (only four individuals). Other species had much lower relative importance, frequency, and efficiency values compared to those already mentioned.

This ranking method is one of the key innovations of our study. By using these indices and overall positioning, we can identify the species that contribute most to the pollen flow of the target plant without visiting frequency masking the importance and efficiency of all species in pollen transport. For example, *F. trichocerata*, one of the least sampled species in our study, achieved a good ranking position because most captures occurred on pistillate inflorescences a result that could be obscured in more traditional analyses, such as ecological interaction networks, where it would be overshadowed by species like the two species of *Habralictus*, which did not appear in our ranking.

Previous studies, such as Villa-Galaviz et al. (2023), used a similar structure to estimate the importance of pollinators, combining quantitative metrics (pQ – pollen quantity) with qualitative ones (pF – pollen fidelity), based on the amount and fidelity of pollen transported by each species. In our study, direct palynological analyses were not feasible at the time of the study, which led to the adaptation of this approach to field parameters that reflect pollen transfer potential. Thus, the indices proposed here, relative importance (Imp) and efficiency (Eff), use the distribution of individuals with pollen in pistillate and staminate inflorescences as proxies for the quantitative and qualitative components of pollination, respectively. This formulation maintains the central statistical logic of integrating the two aspects of pollination performance, while offering a practical and objective way to estimate species contributions to pollen flow adjusted to the circumstances that made palynological analyses unfeasible during the study period.

### Implications for Bee Management

Species of the genus *Frieseomelitta* have been recorded on *E. oleracea* inflorescences in the Amazon estuary (Campbell et al. 2018), but this study presents the first record of *F. trichocerata* contributing to açaí pollen flow. *P. ferreirai* and *T. dallatorreana* were previously reported visiting *Euterpe precatoria* (Rech & Absy 2011), however, to the best of our knowledge, this is the first record of these species visiting *E. oleracea*. *T. williana* has also been recorded visiting *E. oleracea* in previous studies (Marques-Souza et al. 1996) and was observed again in our study.

Studies on the impacts of stingless bee management in commercially important crops like açaí (*E. oleracea*) have shown positive results in the eastern Amazon. Muto et al. (2020) recorded a 2.5-fold increase in fruit size and number per bunch after introducing colonies of *Scaptotrigona* aff. *postica*. However, most of the species identified in our study as potential pollinators of *Euterpe oleracea*, including species of the genera *Trigona* and Partamona, exhibit aggressive nest defense behaviors and often depend on complex nesting substrates, such as arboreal termite mounds, which makes the development of large-scale colony management protocols unlikely for commercial plantations.

In this context, the maintenance and restoration of native forest habitats emerge as the most viable strategy to safeguard these pollinator assemblages. Evidence from eastern Amazonian plantations shows that forest cover at the landscape scale consistently increases bee richness and visitation rates, enhances fruit set and final yield, and ultimately drives higher economic returns for growers, whereas the introduction of managed bee colonies may reduce wild bee richness and even negatively affect initial fruit set (Campbell et al. 2023). These findings reinforce that conserving forest remnants is not only a biodiversity strategy, but also a practical and economically sound approach to sustaining pollination services in açaí production systems.

Our findings highlight the crucial role of native bees as pollinators of *E. oleracea* in the central Amazon. Meliponini was the most influential group in pollen flow for this crop, particularly *T. williana*, *T. dallatorreana*, *F. trichocerata*, and *P. ferreirai*, identified as the main contributors due to their high frequency, importance, and efficiency in pollen transfer between inflorescences. Beyond advancing ecological knowledge, these results provide a practical basis for promoting sustainable management strategies that integrate the conservation of native stingless bees into açaí production systems. Such approaches can enhance pollination services, improve fruit yields, and strengthen the long-term resilience and profitability of açaí cultivation in Amazonas state.

## Data Availability

The datasets generated and/or analyzed during the current study are available from the corresponding author upon reasonable request.
